# The Network of National COVID-19 Data Portals: public health equity through collaboration

**DOI:** 10.12688/openreseurope.20059.1

**Published:** 2025-07-23

**Authors:** Marianna Ventouratou, Liane Hughes, Katarina Öjefors Stark, Johan Rung, Natalia Koralewska, Marzena Ciechomska, Krzysztof Kurowski, Cezary Mazurek, Eva Alloza, Alberto Hornos-Vidal, Salvador Capella-Gutierrez, Robin Navest, Erik Hjerde, Aitana Neves, Colman O'Cathail, Alfonso Valencia, Henning Hermjakob, Matt Pearce, Manish Kumar, Nadim Rahman, Carla Cummins, Guy Cochrane

**Affiliations:** 1 European Molecular Biology Laboratory, European Bioinformatics Institute, Wellcome Genome Campus, Hinxton, Cambridge, CB10 1 SD, UK; 2 SciLifeLab, Stockholm, Stockholm County, Sweden; 3 Institute of Bioorganic Chemistry, Polish Academy of Sciences, Poznan, Poland; 4 National Institute of Geriatrics, Rheumatology, and Rehabilitation, Warsaw, Poland; 5 Poznan Supercomputing and Networking Center, Poznan, Poland; 6 Barcelona Supercomputing Center, Barcelona, Spain; 7 Spanish National Bioinformatics Institute, Barcelona, Spain; 8 Lygature, Utrecht, Utrecht, The Netherlands; 9 The Arctic University of Norway, Tromso, Norway; 10 Swiss Institute of Bioinformatics, Geneva, Switzerland; 11 ICREA, Institucio Catalana de Recerca i Estudis Avancats, Barcelona, Catalonia, Spain

**Keywords:** COVID-19, SARS-CoV-2, FAIR data, COVID-19 Data Platform, COVID-19 Data Portal, National Data Portals, pandemic preparedness, pathogens, infectious diseases, open data

## Abstract

The network of the national COVID-19 Data Portals was developed and linked to the COVID-19 Data Portal ( https://www.covid19dataportal.org/) in response to the need for rapid data sharing and analysis during the 2020-2022 SARS-CoV-2 pandemic. Built on open-source code developed by the Swedish COVID-19 Data Portal (now the Swedish Pathogens Portal, www.pathogens.se) the network included 12 national portals addressing demand for local open data sharing and access, across data types and resources. It provides a robust case study of national initiatives for FAIR (Findable, Accessible, Interoperable and Reusable) resources and a foundation for future pandemic preparedness across pathogens globally. In this paper we outline the structure of the origins of the network of National COVID-19 Datal Portals, the technical aspects and code originating from the Swedish Portal and provide an overview of the services and tools offered by each Portal. The paper showcases the process and operation of four Portals: Sweden, Poland, Spain, Norway and The Netherlands. It considers useful lessons and approaches for future pandemic preparedness which enable researchers to easily identify and obtain the key data from resources on a national and international level.

## Introduction

The European COVID-19 Data Platform was established in April 2020 with funding from the European Commission provided through existing Horizon 2020 projects ^i^ aiming to bring together different services and infrastructures to facilitate sharing of global biodata relevant to SARS-CoV-2. The Platform comprises three components: The COVID-19 Data Portal, the SARS-CoV-2 Data Hubs, and the Federated European Genome Phenome Archive.

The COVID-19 Data Portal ( Harrison *et al.*, 2021) presents a wide range of biological and other data from a number of databases. These include viral and host sequences to scientific articles, images, protein networks, many other molecular data, and social sciences data relating to the pandemic. All data, and in particular the viral sequences, saw a rapid increase in volume as the months progressed ^ii^, currently at ~8.8 million sequences, ~7.1 million raw reads and ~5.6 million systematic analyses. The Portal also offers access to analysis tools and visualisations of viral data, and provided monthly variant reports for SARS-CoV-2 ^iii^ (2021–2023). Fostering open access collaboration and FAIR ^iv^ data sharing, the Portal includes a substantial ‘Related Resources’ section pointing to external databases, studies and resources related to COVID-19 and covering epidemiology, data standards, computing support, tools and cohort data, many linking back to the FAIRsharing catalogue ^v^.

**Figure 1.**
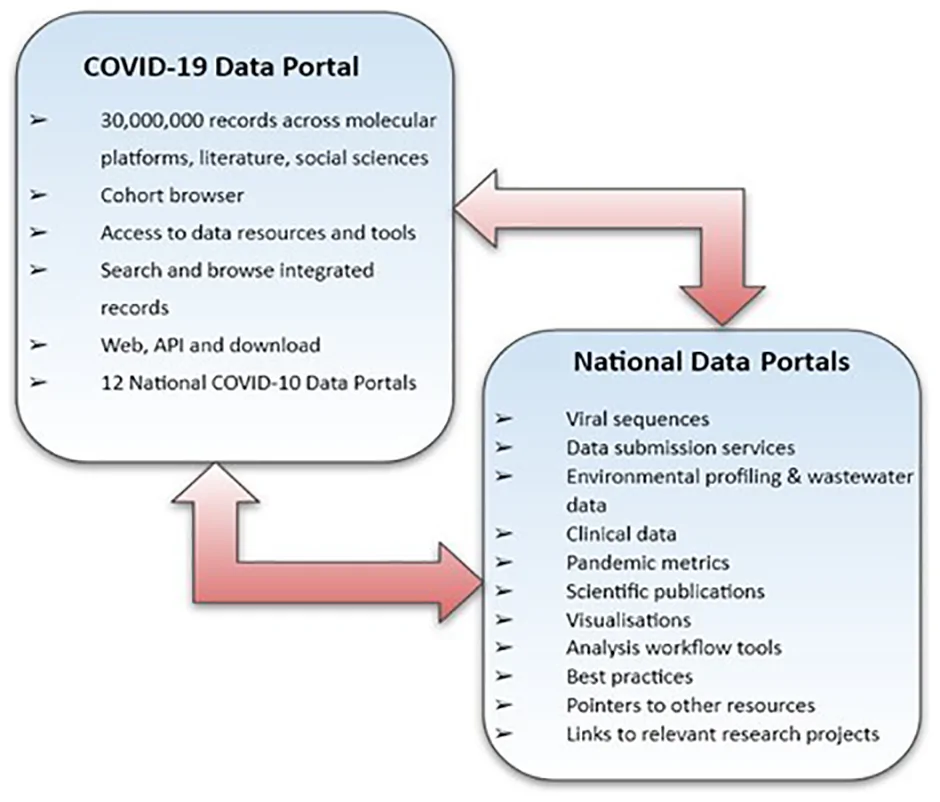
The COVID-19 Data Portal and the National Data Portals: resources and services.

Early in 2020, the notion of National Data Portals to present national level information and links to the COVID-19 Data Portal was developed, which resulted in a network of now 12 bespoke national Portals built over three years ( Figure 1). This article describes the evolution of the network, why and how the Portals were built, what they offer to the scientific community, and which technologies were used. It also aims to capture the lessons learnt from this project and cover plans for the future. Finally, it presents detailed country-specific examples from Sweden, Poland, Spain, Norway and The Netherlands.

## Review of the National COVID-19 Data Portals

### The case for National COVID-19 Data Portals

The National Data Portals provide an important complement to the COVID-19 Data Portal. They offer the scientific community accessibility, visibility and discoverability of national and international data. They also enable further understanding of transmission, infection and symptoms of SARS-CoV-2, and thus aid in the development of treatments, vaccines and public health responses. The National Data Portals offered another layer of visibility for the European COVID-19 Data Platform, which is interlinked through them. The National Data Portals also presented country-specific data and resources in the national language, as well as national health data, epidemiological statistics and links to national studies and other resources; serving thus as an entry point into national resources with appropriate global context.

### Organisation

The network of National Data Portals underlines the interconnectedness of the data across national boundaries and makes the case for data sharing and openness along FAIR principles. It emerged naturally through early, systematic engagement of the COVID-19 Data Platform team at EMBL-EBI in 2020 with the so-called "national coordinators" group, an *ad hoc* community of stakeholders drawn from ELIXIR and EMBL member states and other countries. The national coordination meetings centred on topics around viral data management, brokering, sensitive data management, and the wider, as well as national, clinical and epidemiological context during the early months of the pandemic.

The 12 COVID-19 National Data Portals were configured partly by ELIXIR Nodes and EMBL Member States with the support of EMBL-EBI, which provided the Portal’s graphic templates, and coordinated wider publicity and engagement efforts.

**Figure 2.**
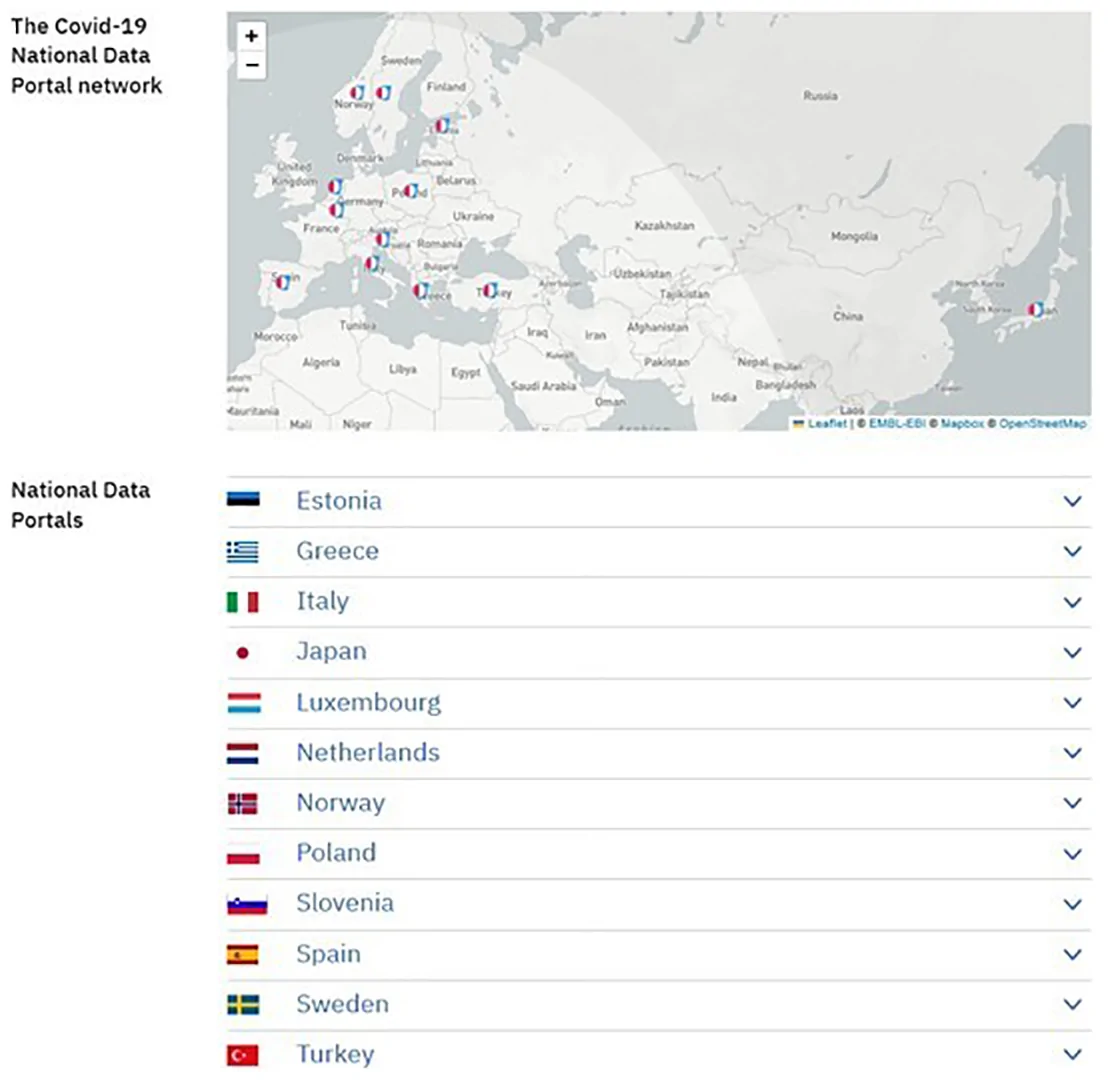
A screenshot of the map and list of National Data Portals on the COVID-19 Data Portal. Source: www.covid19dataportal.org.

Within the first year of the operation of the COVID-19 Data Platform, and until April 2021, Sweden, Poland, Spain, Norway, Italy, Japan, Slovenia and Turkey had set up their national portals. These were followed by Estonia and Greece in autumn 2021. Luxembourg and The Netherlands were added in February and October 2023, respectively ( Figure 2).

### Technical aspects

The first national COVID-19 Data Portal was developed by Sweden. Development started in April 2020, and the code was first made available on GitHub ( Hughes *et al.*, 2024b) in May 2020 under the MIT licence Open Source Initiative ^vi^. The Swedish COVID-19 Data Portal ^vii^ ( SCR_024866) (now known as the Swedish Pathogens Portal, www.pathogens.se) was subsequently launched in June 2020. The portal was specifically designed to enable a quick launch. To facilitate this, the code was written in accessible formats (e.g. markdown), an open source static site generator ( Jain, 2022) was used to build the website, and hosting was configured so as not to require a database and to be effected using any platform. The code has since been reused multiple times to implement other national COVID-19 Portals, and other types of websites. Dynamic elements have been added to the code to ensure that the static nature of the site did not prevent frequent data updates. For example, dynamic data visualisations were produced using Python to enable data to be updated in near real-time (code available on GitHub ^viii^ for reuse under the MIT licence Open Source Initiative ^ix^). Dynamic elements also enable linkage to the European and other national portals ^x^. More information about the technical setup for the Swedish Portal can be found in Hughes *et al.* (2024a).

## National Data Portals features

### Overview

The data displayed on the COVID-19 Data Portal were added based on user requirements and an increasing variety of SARS-CoV-2 related data submissions, first as an urgent response, and eventually as an established project acting as a pilot for future pandemic preparedness. Data and services offered by most National Data Portals set up by January 2022 were diverse ( Table 1). Most portals presented viral sequences, related publications, active research projects and pointers to other resources. A small number of portals offered data submission services, analysis workflow tools and pandemic metrics. Other portals included environmental profiling, including wastewater data, clinical data, visualisations and best practices.

**Table 1.** Overview of the features of the original ten national data portals as of January 2022, and The Netherlands as of October 2023.

	Countries										
Features/Services	EE	GR	IT	JP	NO	PL	SI	ES	SE	TR	NL
*Viral sequences and pointers to viral sequences*		✔	✔	✔	✔	✔		✔	✔		
*Data submission services*	✔			✔	✔	✔	✔		✔		✔
*Environmental profiling/ Wastewater data*	✔	✔					✔		✔		✔
*Clinical data*								✔			
*Pandemic metrics*						✔	✔	✔	✔	✔	✔
*Scientific publications*	✔	✔		✔	✔		✔	✔	✔	✔	✔
*Visualisations*						✔		✔	✔	✔	
*Analysis workflow tools*		✔				✔		✔			
*Vaccination information*						✔	✔		✔		✔
*Best practices*		✔	✔		✔			✔	✔		✔
*Pointers to other resources*	✔		✔	✔		✔	✔	✔	✔	✔	✔
*Research projects*	✔		✔			✔	✔	✔	✔		

*Countries: EE-Estonia, GR-Greece, IT-Italy, JP-Japan, NO-Norway, PL-Poland, SI–Slovenia, ES-Spain,SE-Sweden, TR-Turkey, NL-The Netherlands

This variability in the services and features offered nationally reflects the diverse response to national mandates across Europe while retaining interoperability. This model is strong in that it allowed national frameworks to focus on data and projects that mattered on a national level, while still being deeply connected to the more comprehensive European wide portal. Mobilising, collecting and presenting information on a national level, while a burden on national data providers and coordinators amidst the peaks of the pandemic in 2020 and 2021, demonstrates the resilience of the model to be replicated for pandemic preparedness response.

### Country inputs and descriptions of individual Portals

***Sweden.*** In April 2020, SciLifeLab received an assignment from the Swedish government to accelerate COVID-19 research by helping mobilise and promote relevant data from Sweden. To this end, Sweden launched the first national COVID-19 Data Portal in June 2020 with the express aim of making COVID-19 data more open and FAIR. The portal was developed with the express requirement that it would be quick to launch. This was crucial in ensuring that the portal could be established as a central resource around which the community could aggregate in an emergency situation, such as a global pandemic ( Hughes *et al*., 2024a). To this end, the Swedish Portal was initially developed using volunteers from the Swedish research community, and the code was made openly available to enable other, similar portals to launch quickly (see technical set up section). A dedicated team, composed of individuals with relevant technical expertise and backgrounds in life sciences, was established in Autumn 2024.

The Swedish Portal was embedded in the Swedish research community immediately upon launch. Initial funding for the project was provided by The Swedish Research Council (SRC, Vetenskapsrådet) as part of their wider efforts related to COVID-19. The Swedish Research Council directed other projects in receipt of funding as part of those efforts to contact the Swedish Portal for advice on data management and with help in disseminating their work. SciLifeLab also directed those within their community that were working with COVID-19 to collaborate closely with the portal. This led to close collaborations with the research community, with the portal providing direct support to over 250 research efforts within the first two years. This included work with national organisations (e.g. Biobanks Sweden), national infrastructure (e.g. National Pandemic Centre, and the SciLifeLab Autoimmunity and Serology Profiling unit), and many research groups.

Feedback from the community was sought continuously to ensure that the content of the Swedish Portal met the rapidly evolving needs of the Swedish research community. Priority was given to resources that were considered most broadly useful to the research community, novel research methods that progressed a field, and/or where the resources were not available elsewhere. This led to the development of unique content types. The most popular of these were data highlights (short articles promoting research that shares data, code, and/or tools as openly as possible), data dashboards (pages with descriptions of projects and dynamic data visualisations that are regularly updated with new data), and databases with information about accessing biobanks samples, available research data, and COVID-19 publications from Sweden. As a result, the portal became very popular; receiving over 70,000 unique visitors and 230,000 pageviews in the first two years. Though visitors were primarily from Sweden, there were also frequent visits from countries all around the world.

In order to continue to meet the needs of the Swedish research community, the scope of the portal was expanded in 2022 to reflect a shift in focus towards general pandemic preparedness. The portal became a central pillar of SciLifeLab’s Pandemic Laboratory Preparedness (PLP) program in 2022. The portal has since transitioned to become the Swedish Pathogens Portal, making it the first national Pathogens Portal ^xi^. The aims of the portal are largely unchanged, with the central aim being to make data as FAIR and open as possible.

***Poland.*** At the beginning of April 2020, the Ministry of Science and Higher Education designated the Institute of Bioorganic Chemistry, Polish Academy of Sciences (IBCH PAS) together with the affiliated Poznan Supercomputing and Networking Centre (PSNC) as the national hub for the European COVID-19 Data Platform. IBCH PAS was selected for this role due to its extensive expertise in virology, population genomics, and next-generation sequencing, along with PSNC's advanced supercomputing and networking infrastructure, which enables it to handle large volumes of data while ensuring high standards of data security and integrity. Notably, PSNC manages a robust Polish National Research and Education Network PIONIER, that links all Polish scientific and medical centres with supercomputing and data storage facilities, as well as provides connectivity to the European research network GÉANT, facilitating seamless collaboration and data exchange within the country and with other European entities involved in the COVID-19 Data Platform. Additionally, IBCH PAS and PSNC have strong connections with national and European research networks and extensive experience in large-scale infrastructure projects and partnerships (e.g. EOSC, EuroHPC, PRACE, EUDAT, etc.). The core mission of PSNC, which strengthened the development of the hub, is to foster scientific excellence by providing reliable and cutting-edge e-Infrastructure and highly-specialised laboratories. PSNC achieves this goal, particularly through extensive R&D activities related to information and communication technologies and their innovative applications. In collaborative project teams, they draw from the synergy of science, technology, business, and education to tackle the greatest challenges for our future.

Having such extensive expertise and advanced infrastructure, IBCH PAS and PSNC were able to launch the COVID-19 Data Portal Poland ^xii^, also known as COVID-HUB-PL, within weeks of the Ministry’s decision. The initiative was carried out *pro bono* by a dedicated team of up to 15 people at the peak of the pandemic.

The main aim of this initiative was to facilitate open data sharing and analysis, accelerating research on the coronavirus. The genomic data available through the COVID-19 Data Portal Poland were generated in collaboration with eight healthcare or science centres from Poland. These centres either deposited sequencing data directly or collected biological material sequenced at IBCH PAS. Genomic data and the Poland-focused Nextstrain COVID-19 ^xiii^ instance supervised by COVID-HUB-PL team enabled researchers to monitor the spread and evolution of the virus in real-time, providing insights into how different variants emerged and spread geographically. These studies were essential for informing public health strategies, including lockdown measures. Prioritising viral genomics within the COVID-19 Data Portal Poland ensured that the scientific community and central and local government agencies had access to the most relevant and actionable data to combat the pandemic effectively. Indeed, the aggregated COVID-19 statistics available on the COVID-HUB-PL platform as interactive reports for the country's epidemiological situation enhanced transparency, communication, and data-driven decision-making.

In Poland, efforts to mobilise data regarding COVID-19 have encountered numerous challenges, particularly regarding coordination between different ministries (i.e. Ministry of Science and Higher Education overseeing research and scientific institutions, and Ministry of Health overseeing healthcare and medical centers), and legislative obstacles. Various agencies and institutions involved in COVID-19 response efforts maintained separate data silos, complicating efforts to integrate and harmonise data for analysis. The lack of transparent legislation related to data sharing and privacy constrained the willingness to share viral genomic data through the unrestricted data governance model endorsed by the European COVID-19 Data Platform. Furthermore, the COVID-19 Data Portal Poland was created and continued to operate without additional financial support, using IBCH PAS and PSNC own resources and infrastructure.

**Figure 3.**
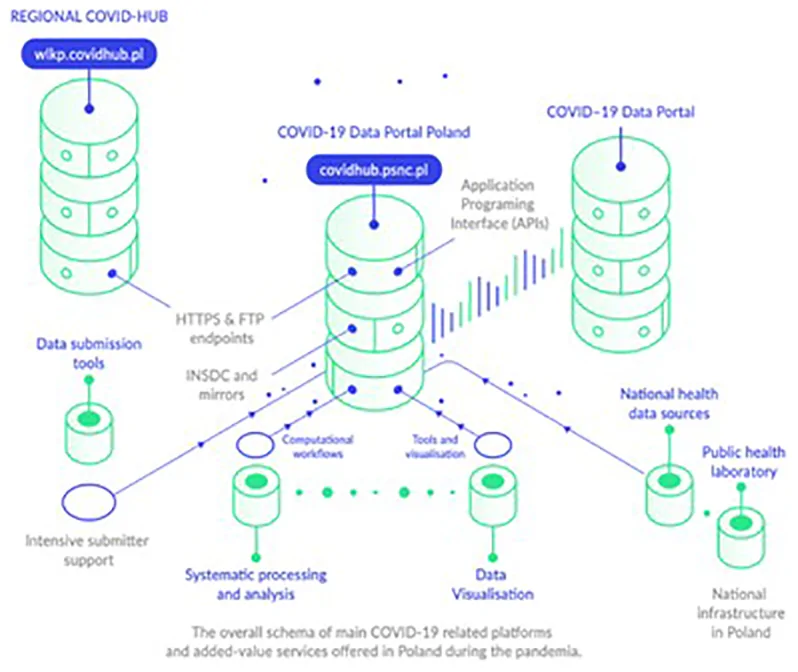
The overall schema of the main COVID-19 related platforms and added value services offered in Poland during the pandemic.

Additionally, the REGIONAL COVID-HUB ^xiv^ platform together with extended e-services have been successfully created thanks to the Wielkopolska Voivodeship Government support and regional WRPO 2014+ programme funding. New and added-value services enabled secure communication and customization for users and public institutions involved in the pandemic response at the Wielkopolska regional level. New capabilities exposed to local users supported advanced visualisation of the pandemic's status and dynamics in Wielkopolska, training and open educational resources for schools as well as functionalities for processing, analysing, and sharing scientific data, and supporting COVID-19 diagnostics using AI methods ( Figure 3).

In the period from 2021 to 2023, the COVID-19 Data Portal Poland had a total of almost 230,000 page views by over 47,000 unique visitors. As of December 2021, the further development of infrastructure and services for the COVID-19 Data Portal Poland has been synchronised with standardisation and best practice exchange activities initiated by the EU ELIXIR-CONVERGE project.

***Spain*** **^xv^**. In the early months of the pandemic, the nodes of the Spanish National Bioinformatics Institute (INB), which represents the Spanish node of ELIXIR (ELIXIR-ES), and the Translational Bioinformatics Network (TransBioNet), held regular meetings to join forces and share knowledge to contribute during the initial uncertainty of the COVID-19 pandemic. One of the actions was the publication in July 2020 of a curated collection of databases, repositories, and tools that were being developed and adapted, as well as publications, projects, and consortia in which they were involved. In parallel, a European effort began through the coordinated work of EMBL-EBI and ELIXIR to create a European portal for the data sharing and analysis of COVID-19 data, the European COVID-19 Data Portal. Spain joined the initiative of tailored national portals after openly publishing the Swedish portal repository.

INB/ELIXIR-ES, as the bioinformatics technology platform of ISCIII in 2020, received a mandate to develop the Spanish portal from ISCIII, the National funding body for Health Research in Spain. This portal was created in the context of the programme *Fondo COVID-19*, an ISCIII emergency funding action to contribute to improving the diagnosis and clinical management of patients infected with SARS-COV-2, the treatment of the disease in the COVID-19 pandemic also contribute to the design, development, and implementation of public health measures to effectively respond to the ongoing SARS-CoV-2 epidemic.

The Spanish COVID-19 Data Portal ^xvi^ showcases Spanish research efforts and technological developments related to SARS-CoV-2 and COVID-19 within the context of the INB/ELIXIR-ES and TransBioNet, and ensures interoperability with other European and international efforts. Thus, the portal highlights a curated compilation of new or adapted data management and analysis tools developed by Spanish researchers. Data and other resources from global repositories are available in the portal, filtering previously Spain as a country and/or affiliation.

Two unique resources were key in decisive moments of the pandemic by offering interactive COVID-19 incidence map and SARS-CoV-2 variant distribution. Firstly, Flow-Maps ^xvii^ ( https://doi.org/10.1038/s41597-021-01093-5), a system for monitoring COVID-19 outbreaks and mobility-associated risk by integrating health information and population-level mobility patterns into a Geographical Information System. Secondly, COVID-19 Viral Beacon, is a tool to find SARS-CoV-2 variability at genomic, amino acid, and motif levels along with other metadata. This implementation was based on the GA4GH Beacon standard and showcased a particular use case for testing and demonstrating the new features of Beacon v2.

**Figure 4.**
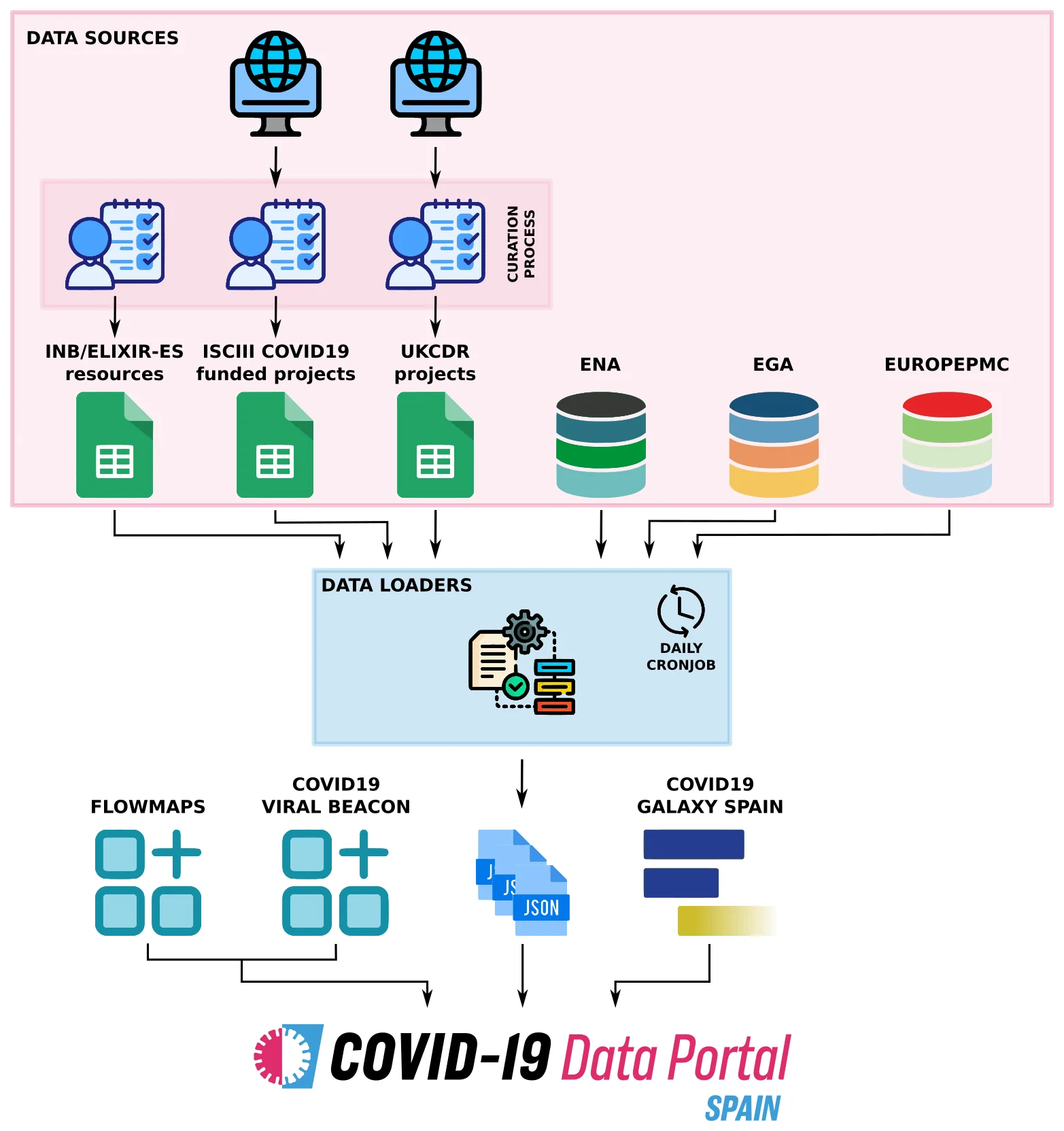
Data flow and presentation schema on the Spanish COVID-19 Data Portal.

In the context of the *Fondo COVID-19*, a significant effort was made to access, normalise and harmonise - following the OMOP Common Data Model - the data generated for the data-driven projects in the programme. An initial set of 60 projects was identified, although the standardised effort was finally carried out for 13 projects after establishing a general procedure for translating individual projects’ codebooks into unambiguous clinical variables, ideally following established ontologies. When unambiguous assignment of variables was not possible, dedicated sessions were carried out with the projects’ data managers. In the rare cases that disambiguation was not possible, concepts were left out of the overall standardisation effort. Once a project was normalised, an overall harmonisation step was performed to ensure general consistency across all datasets provided. Fondo COVID-19 was highlighted by adding filters in relevant sections, e.g. research projects and literature ( Figure 4).

The project was fast developed in three months by a part-time senior full-stack developer and a part-time community engagement and content creation manager. The portal is based on the Swedish portal's open-source code. The first release of the website was launched in December 2020, and it has been in a stable maintenance phase since then, with most of the data updates carried out automatically and certain progressive developments, when necessary, e.g., adding new resources. The technological development of the platform was not a challenge, but the strategic side of it when curating the most significant resources to showcase on our portal.

The Spanish COVID-19 Data Portal has tracked statistics with Google Analytics since March 2021. From March 1st, 2021, until March 1st, 2024, the portal had 5,035 unique visitors, with 31% of the users connecting from Spain, 13.6% from the USA and 6% from the UK. There were 15,279 pages viewed, and 9,722 users engaged. The most visited pages after the home were the literature and genomics sections, followed by the resources page.

**Figure 5.**
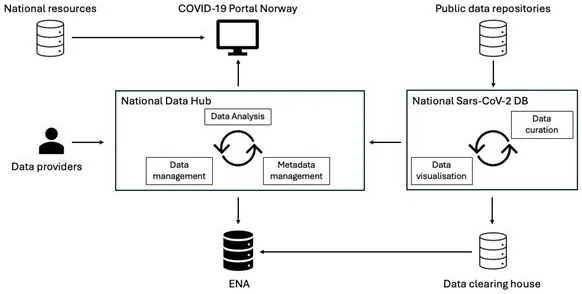
The organisation of data flow and tools and services provided through the Norwegian COVID-19 National Data Portal.

***Norway.*** ELIXIR Norway plays a central national role in giving support on data management of life science data. This includes managing, analysing and sharing sequences, which became essential during the COVID-19 pandemic. The national COVID-19 portal was set up to help and guide researchers and other interested parties. The portal highlights bioinformatics resources such as data sets and analysis methods that were relevant to the situation ( Figure 5). It also is a contact point into the national ELIXIR support desk that could assist researchers wanting to use bioinformatics resources or needed support and advice.

Together with the National Institute of Public Health, the national SARS-CoV-2 data providers were mapped. Through the ELIXIR CONVERGE project, agreements on data quality and contextual metadata standards were implemented nationally. The content of the portal and linking relevant resources was mainly done by ELIXIR Norway personnel. Due to the urgency of the situation at the time and limited funding, no editorial steering group was established.

The portal was set up within months as we based the technology and layout on already existing and open shared source code from other portals in Europe. The Norwegian portal has since transitioned to a pathogen portal ^xviii^ (please see the next section).

***The Netherlands.*** The aim to set up the Dutch national COVID-19 Data Portal ^xix^ was to support researchers and health care professionals with tools and services in their search for ways to overcome the pandemic and its health consequences. The coordination of this national COVID-19 Data Portal was led by Lygature. Other national stakeholders, such as the different university medical centres, Health-RI, RIVM, KNAW/DANS and ODISSEI were involved for specific input and to review content. As there was already a European portal which brings together relevant COVID-19 datasets and tools, we focussed on national resources that are not available through the European platform and thus augment this resource.

All data and resources related to COVID-19 were included with the idea that anything that could be relevant should be available to a researcher or health care professional. To organise this, the Dutch national COVID-19 Data Portal was developed in synergy with the Dutch national resources page on the Infectious Disease Toolkit (IDTk) ^xx^. It was decided to collect information on relevant national health authorities, initiatives and resources on the IDTk national page and focus the portal on COVID-19 relevant dashboards, such as wastewater surveillance, vaccine administration, infection radar and pathogen surveillance.

With the Github example provided by the Swedish portal team, it was easy to create a first version of the portal within a couple of weeks by one person working on it part- *time.* The sustainability of the portal has been the largest challenge. Currently we have ensured a long-term solution for hosting of the portal, but discussions about the maintenance after the BY-COVID project ends are ongoing.

In the period 1 October 2023 until 1 May 2024 the Dutch national COVID-19 Data Portal was visited 4,796 times by 2,040 unique user agents. In this period the main (3,118 visits), contact (251 visits) and about (121 visits) pages were most visited. The vaccine administration in the Netherlands (110 visits) was the most visited data dashboard and imaging data (99 visits) is the most visited data type page.

The Dutch pathogens portal https://www.pathogensportal.nl/ has replaced the national COVID-19 Data Portal. Furthermore, to support stakeholders in finding and gaining access to COVID-19 related datasets, all COVID-19 datasets have been included in the national metadata catalogue https://catalogus.healthdata.nl/?q=COVID.

## Where next with the national portals?

With pandemic preparedness a pressing priority for all countries, the network of National Data Portals is evolving into a network of more generalist national Pathogens Data Portals - treating data and services across broader diseases and pathogens. Receiving a combination of national funding and support from the BY-COVID project ^xxi^, the national Portals are following the path of the Pathogens Portal (link) that has emerged from the COVID-19 Data Portal. Establishing a network of national Pathogens Data Portals will enable access to biodata for pandemic preparedness, with a mixture of pointers to the central Pathogens Portal and own national and regional resources.

The Swedish Pathogens Portal was the first national node, launching just one month after the Pathogens Portal, in August 2023. The existing code base for the Swedish COVID-19 Data Portal was used as a basis and modified to e.g. change the visual identity of the site and add new features related to the expanded scope. The code is available on GitHub ^xxii^ under an MIT licence; not only allowing others to reuse the code to create other national Pathogens Portal but also enabling existing national COVID-19 Portals to transition, should they choose to do so.

Norway and Switzerland have also received funding from the BY-COVID project to set up National Pathogens Portals:

- SIB Swiss Institute of Bioinformatics is currently establishing the Swiss Pathogens Portal to serve the research community and provide detailed information on the available resources, data and dashboards in the field of pathogen bioinformatics and molecular epidemiology. After investigating and exchanging with the SciLifeLab Data Centre team, it was decided to re-use the Swedish Pathogens Portal code base, to also be able to seamlessly benefit (mutually) from future improvements and developments. Α first version of the Swiss portal ^xxiii^ was released in September 2024.
- ELIXIR Norway, represented by The Arctic University of Norway, UiT established the Norwegian Pathogen Portal ^xxiv^ in September 2024. Aiming to connect the portal to a data hub in the future, the team decided to use SvelteKit to develop the web portal. Some parts of the web pages would be rendered via Blazor, while more dynamic pages like dashboards would be rendered by a front-end library like Reacta and SvelteKit. For consistency with the other pathogen portals, we are choosing a design and layout like the Swedish Pathogens Portal. Currently we are mapping the national landscape and are creating content that will become a resource for stakeholders involved in pathogen research and surveillance. The first version was launched September 2024, and there is a pending application with the national research council for funding to extend this work.

A global consortium funded by the NIH NIAID ^xxv^ (award U24AI183840) will continue developing reference software implementation for local pathogens portals (LPPs) and provide support to a growing distributed network of LPPs through seed money and a Community of Practice. The project will also enable better interfacing of the LPPs to the central Pathogens Portal and defining new features bottom-up, driven by the community. The project is motivated by the acknowledgement that accessing and sharing infectious disease data globally is crucial for pandemic preparedness, but obstacles, such as lack of trust and siloed data, can be overcome through local support for analysis, data management and sharing services which are tailored to the local needs.

## Conclusions

This article describes the development of the network of COVID-19 National Data Portals that emerged during the pandemic from 2020 to 2022. It highlights the efforts of specific countries, such as Sweden, Poland, Spain, Norway and The Netherlands. The national portals were all set up to link back to the main COVID-19 Data Portal, a component of the European COVID-19 Data Platform. The portals thus increased the visibility, findability and accessibility of national and international data. The data and resources, covering local and national needs primarily, presented on the portals ranged from viral sequences, data submission services, pandemic metrics, publications and best practices, among others.

In the past year, several of the portals expanded to cover pathogens in general, going beyond SARS-CoV-2 related data, thus creating a springboard for a network of Pathogens Portals. This network will be supported globally by the newly funded NIH project, Pathogen Data Network (award U24AI183840) which has the ambition to create a network of local pathogens portals (LPPs).

The network of COVID–19 national data portals is a testament to the swift and timely efforts of the scientific community to make pandemic-related data available and findable globally. It is also the foundation for any future efforts around outbreak preparedness.

## Data Availability

No data is associated directly with this paper. All datasets supporting the National COVID-19 Data Portals and the COVID-19 Data Portal are available without restrictions from public data repositories, listed here: https://www.covid19dataportal.org/database-resources.

## Software availability

Software developed is described in the article is available from the following:

Source code available from:

https://github.com/ScilifelabDataCentre/pathogens-portal; 10.5281/zenodo.10629602

https://github.com/ScilifelabDataCentre/pathogens-portal-visualisations

https://github.com/ScilifelabDataCentre/pathogens-portal-scripts/tree/main/EBI_indexing

Archive software available from: https://zenodo.org/records/15489081

Licenses:

https://opensource.org/license/mit

https://github.com/ScilifelabDataCentre/pathogens-portal?tab=MIT-1-ov-file#readme

The RRID for the Swedish Pathogens Portal is SCR_024866
