# Supplementary material for: The Network of National COVID-19 Data Portals: public health equity through collaboration
Source: Open Res Eur. 2026 Aug 10;5:192. Originally published 2025 Jul 23. [Version 2] doi: 10.12688/openreseurope.20059.2 (PMC13560843; doi:10.12688/openreseurope.20059.2)
Supplement: Peer review history

# Reviewer report: Reviewer response for version 2

**DOI:** 10.21956/openreseurope.25296.r79142

**Published:** 2026-08-24

**Authors:** Chizaram A. Onyeaghala

**Affiliations:** 1 University of Port Harcourt Teaching Hospital, Port Harcourt, Nigeria

My recommendation

 Recommendation: Approved with reservations — minor/moderate revisions required before indexing.

 The remaining issues I would raise

 1. The paper needs a clearer methodological identity.

 It currently says “In this study, we observe…” but it is not clear whether this is:

- a descriptive case study;
- a technical implementation report;
- a network/infrastructure evaluation;
- a qualitative case study; or
- a narrative description of the portals.

The authors should explicitly state the study/design and explain how the information about the 12 portals and the five showcased portals was collected and analysed.

 This is probably the most important remaining issue.

 2. The title makes a stronger equity claim than the evidence appears to demonstrate.

*The Network of National COVID-19 Data Portals: public health equity through collaboration*

 The manuscript appears to demonstrate collaboration, data sharing, infrastructure development, and FAIR principles much more directly than it demonstrates *public health equity*.

 The authors should either:

- provide evidence demonstrating an equity benefit, such as improved access to data among underserved researchers, institutions or countries; or
- moderate the title and conclusions.

For example:

 The Network of National COVID-19 Data Portals: collaborative infrastructure for equitable access to public health data

 would be more defensible if equity is an important conceptual objective.

 3. The relationship between the 12-portals network and the five showcased portals needs to be clearer.

 The abstract says the network included 12 national portals, but then says the paper showcases Sweden, Poland, Spain, Norway and The Netherlands.

 The manuscript should explain:

- why these five were selected;
- whether they are representative;
- what criteria were used for selection;
- what information was available for the remaining seven portals.

Otherwise, the five examples could inadvertently give the impression that they represent the entire network.

 4. The contribution needs to go beyond description.

 This is an important point for indexing.

 The paper should answer more explicitly:

 What does this case study teach us that was not already known about pandemic data infrastructure?

 The strongest contribution appears to be that pre-existing national infrastructure, interoperable technical architecture, institutional collaboration and sustained investment allowed the portals to be rapidly mobilised during COVID-19.

 That should be elevated as the central analytical finding rather than allowing the paper to become primarily a catalogue of portal features.

 5. “FAIR” should be demonstrated rather than simply invoked.

 The manuscript refers to FAIR resources, but the authors should explain how the portals actually supported:

- Findability;
- Accessibility;
- Interoperability; and
- Reusability.

A concise comparative table would be very useful here.

 6. Sustainability claims need more evidence.

 The conclusion that:

 “Collaborations and preparation built on solid foundations over several years… is key for sustainability…”

 is plausible and probably correct, but the manuscript should distinguish between:

- what the authors observed;
- what they infer; and
- what they recommend for future preparedness.

In particular, the authors should address what happened to the portals after the acute COVID-19 period. Were they maintained, transformed, integrated into broader pathogen-data infrastructure, or discontinued?

 That would substantially strengthen the preparedness argument.

 7. The manuscript should acknowledge limitations of the network as a case study.

 For example:

- selection of participating countries;
- variation in national research infrastructure;
- differences in funding;
- differences in data governance and privacy legislation;
- differences in portal functionality;
- potential selection/reporting bias in the five case examples;
- inability to establish whether portal availability actually improved outbreak decision-making or health outcomes.

This is particularly important given the equity claim.

 8. The terminology needs careful editing.

 There are obvious issues even in the abstract:

- “Datal Portals” → Data Portals
- “origins of the network of National COVID-19 Datal Portals” is awkward.
- “across data types and resources” needs clarification.
- “robust case study” is somewhat evaluative unless supported by explicit criteria.
- “pandemic preparedness across pathogens globally” is broader than the evidence presented.

I would recommend a careful language and terminology edit before acceptance.

Is the rationale for developing the new software tool clearly explained?

Yes

Is the description of the software tool technically sound?

Partly

Are sufficient details of the code, methods and analysis (if applicable) provided to allow replication of the software development and its use by others?

Yes

Is sufficient information provided to allow interpretation of the expected output datasets and any results generated using the tool?

Yes

Reviewer Expertise:

emerging infectious diseases, outbreak response preparedness, and global health.

I confirm that I have read this submission and believe that I have an appropriate level of expertise to confirm that it is of an acceptable scientific standard, however I have significant reservations, as outlined above.

# Reviewer report: Reviewer response for version 2

**DOI:** 10.21956/openreseurope.25296.r78910

**Published:** 2026-08-24

**Authors:** David Horsfall

**Affiliations:** 1 Newcastle University, Newcastle upon Tyne, UK

Thanks for addressing the comments. No further points.

Is the rationale for developing the new software tool clearly explained?

Yes

Is the description of the software tool technically sound?

Partly

Are sufficient details of the code, methods and analysis (if applicable) provided to allow replication of the software development and its use by others?

Yes

Is sufficient information provided to allow interpretation of the expected output datasets and any results generated using the tool?

Partly

Reviewer Expertise:

Research Software Engineering

I confirm that I have read this submission and believe that I have an appropriate level of expertise to confirm that it is of an acceptable scientific standard.

# Reviewer report: Reviewer response for version 1

**DOI:** 10.21956/openreseurope.21697.r64572

**Published:** 2026-01-06

**Authors:** David Horsfall

**Affiliations:** 1 Newcastle University, Newcastle upon Tyne, UK

This article presents a comprehensive account of the conception, development, and operation of a network of National COVID-19 Data Portals that emerged during the SARS-CoV-2 pandemic and were linked to a central European COVID-19 Data Portal. Built initially for the Swedish COVID-19 Data Portal, the network expanded to include twelve national portals, each tailored to local needs. The manuscript provides a compelling case study of how lightweight, reusable infrastructure can support rapid data mobilisation during a public health emergency, while also laying foundations for future preparedness.

 The rationale for developing national portals is clearly explained and well motivated. The authors convincingly argue for the need to complement a central European platform with national entry points that reflect local language, governance structures, data availability, and community trust. The localisation of data and resources within national and regional contexts is a key strength of the work, particularly in overcoming barriers to data discovery and trust that can arise in cross-border settings. The framing of the portals both as an emergency response and as a prototype for longer-term pathogen data infrastructure is effective and timely.

 The technical design choices underpinning the portals are generally sound and well suited to the constraints of rapid deployment, sustainability, and reuse. The use of static site generation with markdown-based content is appropriate and clearly justified. The shared template (originating from the Swedish portal) enabled multiple countries to rapidly deploy functional national hubs that aggregated links to datasets, tools, publications, and services, while signposting users to other national and international resources. The strengths of this approach, particularly speed of deployment and ease of adoption, are clearly articulated, and the reuse of a common codebase appears to have facilitated cross-national collaboration during a period of significant disruption.

 However, the technical description remains largely narrative. A more structured overview of the core components, data flows, and integration points would improve clarity, particularly for technically oriented readers. For example, which elements are shared across portals and which are locally customised would help readers understand how the system scales and evolves.

 While the limitations of a static-site approach are acknowledged, these trade-offs could be explored in more depth. The lack of native dynamic content, database-backed services, or API-level access is an important design constraint, especially given the later discussion of more dynamic web frameworks (e.g. React, Svelte) in the context of future pathogen portals. The manuscript mentions the use of Python-based workflows to generate dynamic data visualisations, and providing a clearer description of how these workflows fit into the overall architecture, would strengthen the technical description.

 The organisational and social dimensions of the work are a notable strength. The description of how the Swedish portal was rapidly embedded within the national research ecosystem (supported by recognition and direction from the Swedish Research Council and SciLifeLab) provides a particularly compelling example of how governance, funding, and infrastructure can align to support community coordination. At the same time, the manuscript highlights that some early efforts relied heavily on volunteer contributions, raising important questions about sustainability and resourcing that are highly relevant for future preparedness initiatives.

 The country-specific case studies are informative and balanced, including constructive discussion of challenges such as data governance barriers, legislative constraints, and the complexities of coordinating across institutions and ministries, as highlighted in the Polish experience. 

 The article provides sufficient information and references to allow replication and reuse by others, particularly through explicit links to GitHub repositories, licensing information, and companion technical publications. 

 The manuscript would benefit from a slightly more systematic treatment of usage metrics and user engagement. While several portals report page views and visitor statistics, the methods used to collect these metrics are not always clear and there is significant variation. Additional context on traffic (how user requirements were gathered in a fast-moving environment, and how feedback from communities was collected and acted upon) would further strengthen the discussion of impact and lessons learned. 

 Overall, this is a valuable and well-documented contribution that captures an important collaborative response to the COVID-19 pandemic. With modest clarification of technical details, and impact interpretation, the manuscript would be further strengthened and serve as a useful reference for future national and international data infrastructure efforts.

Is the rationale for developing the new software tool clearly explained?

Yes

Is the description of the software tool technically sound?

Partly

Are sufficient details of the code, methods and analysis (if applicable) provided to allow replication of the software development and its use by others?

Yes

Is sufficient information provided to allow interpretation of the expected output datasets and any results generated using the tool?

Partly

Reviewer Expertise:

Research Software Engineering

I confirm that I have read this submission and believe that I have an appropriate level of expertise to confirm that it is of an acceptable scientific standard, however I have significant reservations, as outlined above.

# Response

**Published:** 2026-03-05

**Authors:** Marianna Ventouratou

**Affiliations:** EMBL-EBI, Hinxton, UK

*We thank the reviewers for taking the time to provide detailed comments and evaluation of our paper. Please see our individual comments to the reports below.*

 However, the technical description remains largely narrative. A more structured overview of the core components, data flows, and integration points would improve clarity, particularly for technically oriented readers. For example, which elements are shared across portals and which are locally customised would help readers understand how the system scales and evolves.

*We have added a brief explanation, including some limitations, in the section 'Technical aspects’.*

 While the limitations of a static-site approach are acknowledged, these trade-offs could be explored in more depth. The lack of native dynamic content, database-backed services, or API-level access is an important design constraint, especially given the later discussion of more dynamic web frameworks (e.g. React, Svelte) in the context of future pathogen portals. The manuscript mentions the use of Python-based workflows to generate dynamic data visualisations, and providing a clearer description of how these workflows fit into the overall architecture, would strengthen the technical description. 

*An interconnected model with data flows would be an option, but due to funding constraints  and urgency in development, we have not implemented it yet (only partial presentation of covid data from Sweden on the Pathogens Portal for example), There were coordinated data flows which lay outside the Portal network, for example in the  data hubs ( please see here https://www.covid19dataportal.org/data-hubs, and for more details here: https://www.ebi.ac.uk/ena/datahubs/ ). The Portals are more about presentation of data given they flowed into underlying databases. There was coordination, e.g. via the national coordinators, but this was a complementary activity to the portals per se.*

 The organisational and social dimensions of the work are a notable strength. The description of how the Swedish portal was rapidly embedded within the national research ecosystem (supported by recognition and direction from the Swedish Research Council and SciLifeLab) provides a particularly compelling example of how governance, funding, and infrastructure can align to support community coordination. At the same time, the manuscript highlights that some early efforts relied heavily on volunteer contributions, raising important questions about sustainability and resourcing that are highly relevant for future preparedness initiatives.

*We explained the limitations, especially around funding and sustainability in the ‘lessons learnt’ section.*

 The manuscript would benefit from a slightly more systematic treatment of usage metrics and user engagement. While several portals report page views and visitor statistics, the methods used to collect these metrics are not always clear and there is significant variation. Additional context on traffic (how user requirements were gathered in a fast-moving environment, and how feedback from communities was collected and acted upon) would further strengthen the discussion of impact and lessons learned.  

*Usage metrics were requested in 2021–2022 but responses were inconsistent or limited. Through EU-funded projects, the portals and network engaged with community members to gather input, and different groups (e.g. SPSP) contributed requirements from their own networks to guide development. Collecting usage statistics has been challenging due to data availability, external ownership, and possible GDPR constraints, suggesting a need to define clearer, useful impact metrics for the future.*

# Response

**Published:** 2026-07-23

**Authors:** Marianna Ventouratou

**Affiliations:** EMBL-EBI, Hinxton, UK

We thank the reviewer for taking the time to provide detailed comments and evaluation of our paper. Please see our individual comments to the points raised below. However, the technical description remains largely narrative. A more structured overview of the core components, data flows, and integration points would improve clarity, particularly for technically oriented readers. For example, which elements are shared across portals and which are locally customised would help readers understand how the system scales and evolves.

- We have added a brief explanation , including some limitations, in the section 'Technical aspects’. 

  While the limitations of a static-site approach are acknowledged, these trade-offs could be explored in more depth. The lack of native dynamic content, database-backed services, or API-level access is an important design constraint, especially given the later discussion of more dynamic web frameworks (e.g. React, Svelte) in the context of future pathogen portals. The manuscript mentions the use of Python-based workflows to generate dynamic data visualisations, and providing a clearer description of how these workflows fit into the overall architecture, would strengthen the technical description.   

- An interconnected model with data flows would be an option, but due to funding constraints  and urgency in development, we have not implemented it yet (only partial presentation of covid data from Sweden on the Pathogens Portal for example), There were coordinated data flows which lay outside the Portal network, for example in the  data hubs ( please see here https://www.covid19dataportal.org/data-hubs, and for more details here: https://www.ebi.ac.uk/ena/datahubs/ ). The Portals are more about presentation of data given they flowed into underlying databases. There was coordination, e.g. via the national coordinators, but this was a complementary activity to the portals per se.

The organisational and social dimensions of the work are a notable strength. The description of how the Swedish portal was rapidly embedded within the national research ecosystem (supported by recognition and direction from the Swedish Research Council and SciLifeLab) provides a particularly compelling example of how governance, funding, and infrastructure can align to support community coordination. At the same time, the manuscript highlights that some early efforts relied heavily on volunteer contributions, raising important questions about sustainability and resourcing that are highly relevant for future preparedness initiatives.

- We explained the limitations, especially around funding and sustainability in the ‘lessons learnt’ section.

  The manuscript would benefit from a slightly more systematic treatment of usage metrics and user engagement. While several portals report page views and visitor statistics, the methods used to collect these metrics are not always clear and there is significant variation. Additional context on traffic (how user requirements were gathered in a fast-moving environment, and how feedback from communities was collected and acted upon) would further strengthen the discussion of impact and lessons learned.  

- Usage metrics were requested in 2021–2022 but responses were inconsistent or limited. Through EU-funded projects, the portals and network engaged with community members to gather input, and different groups (e.g. SPSP) contributed requirements from their own networks to guide development. Collecting usage statistics has been challenging due to data availability, external ownership, and possible GDPR constraints, suggesting a need to define clearer, useful impact metrics for the future.

# Reviewer report: Reviewer response for version 1

**DOI:** 10.21956/openreseurope.21697.r64578

**Published:** 2025-12-30

**Authors:** Muskan kapoor

**Affiliations:** 1 Iowa State University, Ames, USA

The mansucript describes the coordination and development of various COVID-19 data portals and demonstrate how this helps in data sharing and standardization. The authors very well describve the next steps with national portals highlighting funding along with usage of FAIR pronciples. However, there are few suggestions to improve the clarity of the paper. 

 First would be how were the FAIR principles evaluated, were they based on original COVID-19 Portal? If yes, did they differ across nations and how the author harmonized them?

 How was metadata/ontologies of different data types handled across different portals? The authors should talk more about how different metadata schemas or ontology were made standard.

 Authors should also mention lessons leanred such that this can be extended beyond COVID-19 to other portals thus increasing the applications of this study.

 Finally, It would be great to see citations feature collabortaive work from these portals. 

 Minor comments:

 - Please provide reference in line: A dedicated team, composed of individuals with relevant technical expertise and backgrounds in life sciences, was established in Autumn 2024.

 - Include collabortaive work citations if any: This included work with national organisations (e.g. Biobanks Sweden), national infrastructure (e.g. National Pandemic Centre, and the SciLifeLab Autoimmunity and Serology Profiling unit), and many research groups.

 -Line: These centres either deposited sequencing data directly or collected biological material sequenced at IBCH PAS. Genomic data and the Poland-focused Nextstrain COVID-19 ^xiii^ instance supervised by COVID-HUB-PL team enabled researchers to monitor the spread and evolution of the virus in real-time, providing insights into how different variants emerged and spread geographically. - were there any metadata schema checks performed?

Is the rationale for developing the new software tool clearly explained?

Yes

Is the description of the software tool technically sound?

Yes

Are sufficient details of the code, methods and analysis (if applicable) provided to allow replication of the software development and its use by others?

Yes

Is sufficient information provided to allow interpretation of the expected output datasets and any results generated using the tool?

Yes

Reviewer Expertise:

working on FAIR principles and standardizing metadata schemas between agriculture and human portals. Also worked on developing metadata schemas, automated data transfer, data visualization, annotaion and analysis pipelines between portals

I confirm that I have read this submission and believe that I have an appropriate level of expertise to confirm that it is of an acceptable scientific standard, however I have significant reservations, as outlined above.

# Response

**Published:** 2026-03-05

**Authors:** Marianna Ventouratou

**Affiliations:** EMBL-EBI, Hinxton, UK

*We thank the reviewers for taking the time to provide detailed comments and evaluation of our paper. Please see our individual comments to the reports below.*

1. First would be how were the FAIR principles evaluated, were they based on the original COVID-19 Portal? If yes, did they differ across nations and how the author harmonized them?

*The European COVID-19 Data Portal presents data that originate from FAIR-compliant repositories within EMBL-EBI and beyond, so the data presented on it adhere to FAIR principles. As the portal primarily aggregates and displays these archived datasets, its FAIR compliance largely reflects that of the underlying repositories. The Swedish Portal is also listed on FAIRsharing, supporting its status as a FAIR-aligned resource.*

*While the national portals may not uniformly meet identical standards and some elements, such as dashboards drawing on national or institutional sources, may vary in implementation, the underlying data are generally sourced from ELIXIR Core Data Resource repositories ( https://elixir-europe.org/platforms/data/core-data-resources; at least 10 of them indexed on the COVID-19 Data Portal) that follow FAIR standards, ensuring overall alignment with FAIR principles.   *  

 2. How was metadata/ontologies of different data types handled across different portals? The authors should talk more about how different metadata schemas or ontology were made standard.

*The national portals didn't really bring together different data types, so they weren't handled 'across portals'. This is something that we are still working towards with the Pathogen Portals using a three-tiered indexing system, where in the lower tier there is just a link out to the Pathogen Portal node (as the ‘national portals’ have been renamed for the Pathogen Portals network). Tier 2, where some of the data is presented on the main Pathogens Portal (e.g. https://www.pathogensportal.org/nationaldataportal?db=national-portals-sweden) to a fully findable Tier 3 where all the data from the nodes is findable and presented on the main Portal.*

 3. Authors should also mention lessons learned such that this can be extended beyond COVID-19 to other portals thus increasing the applications of this study. *A lesson learnt and limitations section has been added.*

 4. Finally, it would be great to see citations feature collaborative work from these portals. 

*These are portals rather than direct databases, so it is more likely that there are publications related to the use of the underlying resources (i.e. the databases), rather than the portals themselves.  *  

 Minor comments:

1. Please provide reference in line: A dedicated team, composed of individuals with relevant technical expertise and backgrounds in life sciences, was established in Autumn 2024. *DONE*
2. Include collaborative work citations if any: This included work with national organisations (e.g. Biobanks Sweden), national infrastructure (e.g. National Pandemic Centre, and the SciLifeLab Autoimmunity and Serology Profiling unit), and many research groups. *DONE*
3. Line: These centres either deposited sequencing data directly or collected biological material sequenced at IBCH PAS. Genomic data and the Poland-focused Nextstrain COVID-19 ^xiii^ instance supervised by COVID-HUB-PL team enabled researchers to monitor the spread and evolution of the virus in real-time, providing insights into how different variants emerged and spread geographically. - were there any metadata schema checks performed?.

*Yes, metadata schema checks were performed. First, contributing centers were provided with standardised Excel templates. Although these templates included multiple predefined fields, in collaboration agreements with our partners, we also defined a minimum mandatory metadata set required for any record to be accepted. Second, upon deposition on our server, each metadata file underwent a validation process by the COVID-HUB-PL team. We checked for completeness, adherence to controlled vocabularies, and format consistency. Any identified discrepancies or missing values were clarified and corrected before they were integrated into the Nextstrain instance or submitted to the databases.*

# Response

**Published:** 2026-07-23

**Authors:** Marianna Ventouratou

**Affiliations:** EMBL-EBI, Hinxton, UK

1. First would be how were the FAIR principles evaluated, were they based on the original COVID-19 Portal? If yes, did they differ across nations and how the author harmonized them?

The European COVID-19 Data Portal presents data that originate from FAIR-compliant repositories within EMBL-EBI and beyond, so the data presented on it adhere to FAIR principles. As the portal primarily aggregates and displays these archived datasets, its FAIR compliance largely reflects that of the underlying repositories. The Swedish Portal is also listed on FAIRsharing, supporting its status as a FAIR-aligned resource.  While the national portals may not uniformly meet identical standards and some elements, such as dashboards drawing on national or institutional sources, may vary in implementation, the underlying data are generally sourced from ELIXIR Core Data Resource repositories ( https://elixir-europe.org/platforms/data/core-data-resources; at least 10 of them indexed on the COVID-19 Data Portal) that follow FAIR standards, ensuring overall alignment with FAIR principles.   

1. How was metadata/ontologies of different data types handled across different portals? The authors should talk more about how different metadata schemas or ontology were made standard.

The national portals didn't really bring together different data types, so they weren't handled 'across portals'. This is something that we are still working towards with the Pathogen Portals using a three-tiered indexing system, where in the lower tier there is just a link out to the Pathogen Portal node (as the ‘national portals’ have been renamed for the Pathogen Portals network). Tier 2, where some of the data is presented on the main Pathogens Portal (e.g. https://www.pathogensportal.org/nationaldataportal?db=national-portals-sweden) to a fully findable Tier 3 where all the  data from the nodes is findable and presented on the main Portal.

1. Authors should also mention lessons learned such that this can be extended beyond COVID-19 to other portals thus increasing the applications of this study. 

A lessons learnt and limitations section has been added.

1. Finally, It would be great to see citations feature collaborative work from these portals. 

These are portals rather than direct databases, so it is more likely that there are publications related to the use of the underlying resources (i.e. the databases), rather than the portals themselves.    Minor comments:

1. Please provide reference in line: A dedicated team, composed of individuals with relevant technical expertise and backgrounds in life sciences, was established in Autumn 2024. DONE
2. Include collaborative work citations if any: This included work with national organisations (e.g. Biobanks Sweden), national infrastructure (e.g. National Pandemic Centre, and the SciLifeLab Autoimmunity and Serology Profiling unit), and many research groups. DONE
3. Line: These centres either deposited sequencing data directly or collected biological material sequenced at IBCH PAS. Genomic data and the Poland-focused Nextstrain COVID-19 ^xiii^ instance supervised by COVID-HUB-PL team enabled researchers to monitor the spread and evolution of the virus in real-time, providing insights into how different variants emerged and spread geographically. - were there any metadata schema checks performed?.

Yes, metadata schema checks were performed. First, contributing centers were provided with standardised Excel templates. Although these templates included multiple predefined fields, in collaboration agreements with our partners, we also defined a minimum mandatory metadata set required for any record to be accepted. Second, upon deposition on our server, each metadata file underwent a validation process by the COVID-HUB-PL team. We checked for completeness, adherence to controlled vocabularies, and format consistency. Any identified discrepancies or missing values were clarified and corrected before they were integrated into the Nextstrain instance or submitted to the databases.

# Reviewer report: Reviewer response for version 1

**DOI:** 10.21956/openreseurope.21697.r57237

**Published:** 2025-12-11

**Authors:** Flavia Bernardini

**Affiliations:** 1 Universidade Federal Fluminense, Rio de Janeiro, Brazil

The work presents an interesting case study of implementing COVID-19 national data ports in 12 countries through the EU. The authors state that these portals form an interconnected network, adhering to FAIR principles. This initiative is quite important, considering both the perspective of implementing good practices for national open data portals and the perspective of establishing and implementing open data ecosystems. Additionally, describing how each data portal evolved as a technical solution is quite interesting, considering the requirements pointed out by the national governments of each country.

 However, the paper lacks the rationale and technical details for developing the new software tool. I explain in detail each aspect of the paper that, from my perspective, is still confusing:

 - The manuscript starts by presenting a network of national COVID-19 Data Portals. As my background is in Computer Science, I tend to imagine these data portals are someway fully connected. For me, if this is the case, the portals also interoperate with one another. In this way, I could expect that if a user searches for data in one portal, depending on the level of interoperability, this search could retrieve data from the other portals in the network. However, after reading the paper, it seems to me that this is not the case. Rather, it seems to me that the portals indeed offer some kind of technology for interoperability (perhaps APIs) to interoperate with them, but I'm not sure. This perspective is reinforced by the technical aspects described in the Review of the National COVID-19 Data Portals section. So, in this sense, the term "network" is more likely to be associated with a conceptual network rather than guaranteeing full interoperability. In this regard, I recommend that the authors clarify these aspects properly.

 - When reading the paper, I remembered the initiative of Common European Data Spaces - https://digital-strategy.ec.europa.eu/en/policies/data-spaces - that aims to serve as a fully integrative platform for open data in Europe. There are some implementations of data spaces in some countries within the EU. Does this described network intend to align with this initiative in the future? 

 - The authors state that "systematic engagement of the COVID-19 Data Platform team at EMBL-EBI in 2020 with the so-called "national coordinators" group, an *ad hoc* community of stakeholders drawn from ELIXIR and EMBL member states and other countries". It seems to me that these organizations were somehow responsible for developing all these national data portals, but I'm not entirely sure. Also, I have no clue if these (EMBL-EBI, ELIXIR, and EMBL) are indeed organizations or what their rule are in defining this network, if this is the case. These "organization" aspects, including the responsibility of each organ and whether or not they are connected to ELIXIR, and if they are, how, are repeated throughout the paper. For instance, when describing the Sweden case, the authors state that "SciLifeLab received an assignment". What is SciLifeLab? This pattern repeats when the authors present other cases from countries such as Poland and the Netherlands, as the Spanish and Norwegian cases suggest that they are somehow related to ELIXIR. Is the network of actors that together define some architectural aspect? I recommend that the authors properly present the relations among all the actors listed in the work. I also suggest that the authors clearly state how these portals are interconnected - through authors in national governments, through a defined architecture to portals, or both?

 - In this part of the manuscript: "This variability in the services and features offered nationally reflects the diverse response to national mandates across Europe while retaining interoperability. This model is strong in that it allowed national frameworks to focus on data and projects that mattered on a national level, while still being deeply connected to the more comprehensive European wide portal", what are the meanings of the word "model" and "framework"? From the perspective of modeling an architecture, framework, process, and so on, it is expected that a model or framework is properly modeled and presented, using some notation if a diagram is used. Did the authors model the network? Is the available software a computational framework?

 I think that, after clarifying these aspects, it will be easier to better understand the software tool the authors are presenting and how to (re)use it to implement Pathogens Data Portals.

Is the rationale for developing the new software tool clearly explained?

Partly

Is the description of the software tool technically sound?

Partly

Are sufficient details of the code, methods and analysis (if applicable) provided to allow replication of the software development and its use by others?

No

Is sufficient information provided to allow interpretation of the expected output datasets and any results generated using the tool?

Yes

Reviewer Expertise:

Computer Science, Data Science, Open Data, Machine Learning and Artificial Intelligence, Data Government

I confirm that I have read this submission and believe that I have an appropriate level of expertise to confirm that it is of an acceptable scientific standard, however I have significant reservations, as outlined above.

# Response

**Published:** 2026-03-05

**Authors:** Marianna Ventouratou

**Affiliations:** EMBL-EBI, Hinxton, UK

We thank the reviewers for taking the time to provide detailed comments and evaluation of our paper. Please see our individual comments to the reports below.  

1. However, the paper lacks the rationale and technical details for developing the new software tool. I explain in detail each aspect of the paper that, from my perspective, is still confusing:

*The technology used varied with many using the Swedish codebase as outlined in the article, but it was not our intention to proscribe and promote a single technical solution.*  

 2. The manuscript starts by presenting a network of national COVID-19 Data Portals. As my background is in Computer Science, I tend to imagine these data portals are someway fully connected. For me, if this is the case, the portals also interoperate with one another. In this way, I could expect that if a user searches for data in one portal, depending on the level of interoperability, this search could retrieve data from the other portals in the network. However, after reading the paper, it seems to me that this is not the case. Rather, it seems to me that the portals indeed offer some kind of technology for interoperability (perhaps APIs) to interoperate with them, but I'm not sure. This perspective is reinforced by the technical aspects described in the Review of the National COVID-19 Data Portals section. So, in this sense, the term "network" is more likely to be associated with a conceptual network rather than guaranteeing full interoperability. In this regard, I recommend that the authors clarify these aspects properly.

*To clarify, we have added an explanation regarding the usage of the word ‘network’ in the last paragraph of the introduction.*  

 3. When reading the paper, I remembered the initiative of Common European Data Spaces - https://digital-strategy.ec.europa.eu/en/policies/data-spaces - that aims to serve as a fully integrative platform for open data in Europe. There are some implementations of data spaces in some countries within the EU. Does this described network intend to align with this initiative in the future?   

*Part of the Common European Data Spaces, under the Health space,  is the European Genomics Data Infrastructure (GDI). Several of the partners of the EGDI were also part of the Horizon Europe projects that supported the European COVID-19 Data Platform. The Pathogen Platform and the Pathogens Portal, which is the natural evolution of the COVID-19 Portals, also involves the same organisations and infrastructures. For example: EMBL-EBI, ELIXIR, BBMRI, Erasmus Medical Center Rotterdam, Barcelona Supercomputing Center (BSC), Carlos III Health Institute (ISCII), Centre for Genomic Regulation (CRG). Hence there are already ongoing synergies, communication and collaboration, all aligning towards the Common European Data Space. These can certainly be exploited further in the future, should the opportunity and the funding become available.*

 4. The authors state that "systematic engagement of the COVID-19 Data Platform team at EMBL-EBI in 2020 with the so-called "national coordinators" group, an ad hoc community of stakeholders drawn from ELIXIR and EMBL member states and other countries". It seems to me that these organizations were somehow responsible for developing all these national data portals, but I'm not entirely sure. Also, I have no clue if these (EMBL-EBI, ELIXIR, and EMBL) are indeed organizations or what their rule are in defining this network, if this is the case. These "organization" aspects, including the responsibility of each organ and whether or not they are connected to ELIXIR, and if they are, how, are repeated throughout the paper. For instance, when describing the Sweden case, the authors state that "SciLifeLab received an assignment". What is SciLifeLab?  This pattern repeats when the authors present other cases from countries such as Poland and the Netherlands, as the Spanish and Norwegian cases suggest that they are somehow related to ELIXIR. Is the network of actors that together define some architectural aspect? I recommend that the authors properly present the relations among all the actors listed in the work. I also suggest that the authors clearly state how these portals are interconnected - through authors in national governments, through a defined architecture to portals, or both?

*For clarity we have spelled out the acronyms for each organisation and institution, added a brief description of each one in the footnotes and added links to their respective websites.*

 5. In this part of the manuscript: "This variability in the services and features offered nationally reflects the diverse response to national mandates across Europe while retaining interoperability. This model is strong in that it allowed national frameworks to focus on data and projects that mattered on a national level, while still being deeply connected to the more comprehensive European wide portal", what are the meanings of the word "model" and "framework"? From the perspective of modeling an architecture, framework, process, and so on, it is expected that a model or framework is properly modeled and presented, using some notation if a diagram is used. Did the authors model the network? Is the available software a computational framework? 

*As the “network of the Portals” is more of an output of social cooperation, the word  “model” is also used in the sense that it means a simplified description of a system, rather than a computational model.*

# Response

**Published:** 2026-07-23

**Authors:** Marianna Ventouratou

**Affiliations:** EMBL-EBI, Hinxton, UK

We thank the reviewer for taking the time to provide detailed comments and evaluation of our paper. Please see our individual comments to the points raised in the review below.   The work presents an interesting case study of implementing COVID-19 national data ports in 12 countries through the EU. The authors state that these portals form an interconnected network, adhering to FAIR principles. This initiative is quite important, considering both the perspective of implementing good practices for national open data portals and the perspective of establishing and implementing open data ecosystems. Additionally, describing how each data portal evolved as a technical solution is quite interesting, considering the requirements pointed out by the national governments of each country.  

1. However, the paper lacks the rationale and technical details for developing the new software tool. I explain in detail each aspect of the paper that, from my perspective, is still confusing:

  The technology used varied with many using the Swedish codebase as outlined in the article, but it was not our intention to proscribe and promote a single technical solution.  

1. The manuscript starts by presenting a network of national COVID-19 Data Portals. As my background is in Computer Science, I tend to imagine these data portals are someway fully connected. For me, if this is the case, the portals also interoperate with one another. In this way, I could expect that if a user searches for data in one portal, depending on the level of interoperability, this search could retrieve data from the other portals in the network. However, after reading the paper, it seems to me that this is not the case. Rather, it seems to me that the portals indeed offer some kind of technology for interoperability (perhaps APIs) to interoperate with them, but I'm not sure. This perspective is reinforced by the technical aspects described in the Review of the National COVID-19 Data Portals section. So, in this sense, the term "network" is more likely to be associated with a conceptual network rather than guaranteeing full interoperability. In this regard, I recommend that the authors clarify these aspects properly.

To clarify, we have added an explanation regarding the usage of the word ‘network’ in the last paragraph of the introduction.  

1. When reading the paper, I remembered the initiative of Common European Data Spaces - https://digital-strategy.ec.europa.eu/en/policies/data-spaces - that aims to serve as a fully integrative platform for open data in Europe. There are some implementations of data spaces in some countries within the EU. Does this described network intend to align with this initiative in the future? 

  Part of the Common European Data Spaces, under the Health space,  is the European Genomics Data Infrastructure (GDI). Several of the partners of the EGDI were also part of the Horizon Europe projects that supported the European COVID-19 Data Platform. The Pathogen Platform and the Pathogens Portal, which is the natural evolution of the COVID-19 Portals, also involves the same organisations and infrastructures. For example: EMBL-EBI, ELIXIR, BBMRI, Erasmus Medical Center Rotterdam, Barcelona Supercomputing Center (BSC), Carlos III Health Institute (ISCII), Centre for Genomic Regulation (CRG). Hence there are already ongoing synergies, communication and collaboration, all aligning towards the Common European Data Space. These can certainly be exploited further in the future, should the opportunity and the funding become available.  

1. The authors state that "systematic engagement of the COVID-19 Data Platform team at EMBL-EBI in 2020 with the so-called "national coordinators" group, an ad hoc community of stakeholders drawn from ELIXIR and EMBL member states and other countries". It seems to me that these organizations were somehow responsible for developing all these national data portals, but I'm not entirely sure. Also, I have no clue if these (EMBL-EBI, ELIXIR, and EMBL) are indeed organizations or what their rule are in defining this network, if this is the case. These "organization" aspects, including the responsibility of each organ and whether or not they are connected to ELIXIR, and if they are, how, are repeated throughout the paper. For instance, when describing the Sweden case, the authors state that "SciLifeLab received an assignment". What is SciLifeLab?  This pattern repeats when the authors present other cases from countries such as Poland and the Netherlands, as the Spanish and Norwegian cases suggest that they are somehow related to ELIXIR. Is the network of actors that together define some architectural aspect? I recommend that the authors properly present the relations among all the actors listed in the work. I also suggest that the authors clearly state how these portals are interconnected - through authors in national governments, through a defined architecture to portals, or both?

For clarity we have spelled out the acronyms for each organisation and institution, added a brief description of each one in the footnotes and added links to their respective websites.  

1. In this part of the manuscript: "This variability in the services and features offered nationally reflects the diverse response to national mandates across Europe while retaining interoperability. This model is strong in that it allowed national frameworks to focus on data and projects that mattered on a national level, while still being deeply connected to the more comprehensive European wide portal", what are the meanings of the word "model" and "framework"? From the perspective of modeling an architecture, framework, process, and so on, it is expected that a model or framework is properly modeled and presented, using some notation if a diagram is used. Did the authors model the network? Is the available software a computational framework? 

As the “network of the Portals” is more of an output of social cooperation, the word  “model” is also used in the sense that it means a simplified description of a system, rather than a computational model. For clarity, we replaced the word model with 'configuration', and frameworks with 'structures'.

# Reviewer report: Reviewer response for version 1

**DOI:** 10.21956/openreseurope.21697.r57662

**Published:** 2025-09-04

**Authors:** Gopi Battineni

**Affiliations:** 1 University of Camerino, Camerino, Italy

Marianna et al developed real time COVID 19 data portal which add the network value of national COVID-19 data portals. I had few suggestions before accepting this work

 The abstract could be improved by highlighting the impact. It reads more like a descriptive summary than an analytical one. The lessons learned from COVID-19 should be clarified.

 Several sentences in the introduction are long and full of references, which makes it difficult to read.

 The inclusion of a critical perspective on coordination challenges, such as governance and sustainability, may be beneficial

 I think supplementary materials would be better suited to cover implementation details (markdown, site generator, hosting).

 Add comments on the most widely used and valuable features in national data portals.

 The section titled "next with national portals" highlighted the transition to pathogen portals, but it is fragmented, with too much emphasis on technical details.

 For pandemic preparedness, it is important to emphasize lessons learned.

 An unresolved issue and limitations paragraph should be added.

 Despite its strengths, the report could have benefited from a clearer discussion of limitations and lessons learned.

Is the rationale for developing the new software tool clearly explained?

Yes

Is the description of the software tool technically sound?

Yes

Are sufficient details of the code, methods and analysis (if applicable) provided to allow replication of the software development and its use by others?

Yes

Is sufficient information provided to allow interpretation of the expected output datasets and any results generated using the tool?

Yes

Reviewer Expertise:

Data mining, machine learning, pedictive analytics

I confirm that I have read this submission and believe that I have an appropriate level of expertise to confirm that it is of an acceptable scientific standard, however I have significant reservations, as outlined above.

# Response

**Published:** 2026-03-05

**Authors:** Marianna Ventouratou

**Affiliations:** EMBL-EBI, Hinxton, UK

We thank the reviewers for taking the time to provide detailed comments and evaluation of our paper. Please see our individual comments to the reports below. 1st Report - Gopi Battineni, University of Camerino, Camerino, Italy

1. The abstract could be improved by highlighting the impact. It reads more like a descriptive summary than an analytical one. 
  1. The lessons learned from COVID-19 should be clarified. 

We added a sentence in the abstract, which is further expanded in the ‘Lessons learnt and limitations’ section.

1. Several sentences in the introduction are long and full of references, which makes it difficult to read.

We rephrased the introduction as much as this was possible.

1. The inclusion of a critical perspective on coordination challenges, such as governance and sustainability, may be beneficial 

We rephrased and expanded the first paragraph under section ‘Organisation’ to address this suggestion.

1. I think supplementary materials would be better suited to cover implementation details (markdown, site generator, hosting).

We have taken this into consideration, this article is intended to provide an overview of the strategic, technical and connectivity-related elements of the various Portals.

1. Add comments on the most widely used and valuable features in national data portals. 

This is a limitation we acknowledge, and difficult to address due the restrictions and variability in statistics held for each Portal. We have added a paragraph to this effect under the ‘Lessons learnt and limitations’.  

1. The section titled "next with national portals" highlighted the transition to pathogen portals, but it is fragmented, with too much emphasis on technical details. 

We revised the wording in this section to provide a more holistic view. 

1. For pandemic preparedness, it is important to emphasize lessons learned.

1. An unresolved issue and limitations paragraph should be added.

1. Despite its strengths, the report could have benefited from a clearer discussion of f limitations and lessons learned.

A special section ‘Lessons learnt and limitations’ has been added to address the reviewer comments related to the lessons learnt and limitations overall.

# Response

**Published:** 2026-07-23

**Authors:** Marianna Ventouratou

**Affiliations:** EMBL-EBI, Hinxton, UK

We thank the reviewer for taking the time to provide detailed comments and evaluation of our paper. Please see our individual comments to the points raised in the review below.

1. The abstract could be improved by highlighting the impact. It reads more like a descriptive summary than an analytical one. 
  1. The lessons learned from COVID-19 should be clarified. 

We added a sentence in the abstract, which is further expanded in the ‘Lessons learnt and limitations’ section.  

1. Several sentences in the introduction are long and full of references, which makes it difficult to read.

We rephrased the introduction as much as this was possible.  

1. The inclusion of a critical perspective on coordination challenges, such as governance and sustainability, may be beneficial 

We rephrased and expanded the first paragraph under section ‘Organisation’ to address this suggestion.

  

1. I think supplementary materials would be better suited to cover implementation details (markdown, site generator, hosting).

We have taken this into consideration, this article is intended to provide an overview of the strategic, technical and connectivity-related elements of the various Portals  

1. Add comments on the most widely used and valuable features in national data portals. 

  This is a limitation we acknowledge, and difficult to address due the restrictions and variability in statistics held for each Portal. We have added a paragraph to this effect under the ‘Lessons learnt and limitations’.  

1. The section titled "next with national portals" highlighted the transition to pathogen portals, but it is fragmented, with too much emphasis on technical details. 

We revised the wording in this section to provide a more holistic view. 

1. For pandemic preparedness, it is important to emphasize lessons learned.

1. An unresolved issue and limitations paragraph should be added.

1. Despite its strengths, the report could have benefited from a clearer discussion of f limitations and lessons learned.

Done; a special section ‘Lessons learnt and limitations’ has been added to address the reviewer comments related to the lessons learnt and limitations overall.
